# Robotic-Assisted Total Knee Arthroplasty Utilizing NAVIO, CORI Imageless Systems and Manual TKA Accurately Restore Femoral Rotational Alignment and Yield Satisfactory Clinical Outcomes: A Randomized Controlled Trial

**DOI:** 10.3390/medicina59020236

**Published:** 2023-01-27

**Authors:** Olga Adamska, Krzysztof Modzelewski, Jakub Szymczak, Jakub Świderek, Bartosz Maciąg, Paweł Czuchaj, Małgorzata Poniatowska, Artur Wnuk

**Affiliations:** 1 Orthopedic and Rehabilitation Department, Medical University of Warsaw, 61 Żwirki i Wigury St., 02-091 Warsaw, Poland; 2 Faculty of Medicine, Medical University of Bialystok, 1 Jana Kilińskiego St., 15-089 Bialystok, Poland; 3 Collegium Medicum, University of Zielona Góra, 28 Zyty St., 65-046 Zielona Góra, Poland; 4 Hospital in Ostrow Mazowiecka, 68 Dubois St., 07-300 Ostrów Mazowiecka, Poland

**Keywords:** CORI, imageless robotic-assisted TKA, NAVIO, OA treatment, osteoarthritis, robotic-assisted TKA, robotic surgery, TKA

## Abstract

*Background and objectives*: The introduction of novel techniques in total knee arthroplasty (TKA) aiming to enhance outcomes and satisfaction of the procedure is constantly ongoing. In order to evidence a priority of one, we have conducted a randomized controlled trial with the aim of comparing patient-reported functional outcomes, radiographic outcomes and intraoperative measures between imageless (NAVIO and CORI), robotic-assisted (ra)- TKA (ra-TKA) and manual TKA (mTKA) for primary knee osteoarthritis (KOA). *Materials and Methods*: A total of 215 patients with the diagnosis of KOA of the knee were randomly assigned to one of the three groups: NAVIO (76 patients) or CORI (71 patients) robotic-assisted TKA, or manual technique (68 patients) TKA. The primary outcome (Knee Injury and Osteoarthritis Outcome Study [KOOS]), Visual Analogue Scale (VAS), Range of motion (ROM), femoral component rotational alignment and the secondary outcomes (surgery time, blood loss, complications, and revision at 12 months after surgery) were compared between three groups. KOOS and VAS were collected at particular follow up visits from each patient individually and ROM in flexion and extension was assessed during the physical examination. Femoral component rotational alignment was measured on the CT scan performed postoperatively utilizing the Berger’s method. Statistical significance was set at *p* < 0.05. *Results*: Both the ra-TKA groups and mTKA group displayed significant improvements in the majority of the functional outcome scores at 12 months. Despite having more prominent surgery time (NAVIO: mean +44.5 min in comparison to mTKA and CORI: mean +38.5 min in comparison to mTKA), both NAVIO and CORI tend to achieve highly accurate femoral component rotational alignment with mean radiographic scores in NAVIO vs. CORI vs. mTKA of 1.48° vs. 1.33° vs. 3.15° and lower blood loss (NAVIO: 1.74; CORI: 1.51; mTKA: 2.32. Furthermore, the investigation revealed the significant difference in femoral component rotational alignment between mTKA—NAVIO and mTKA—CORI and significantly different KOOS scores in NAVIO vs. CORI vs. mTKA of 87.05 vs. 85.59 vs. 81.76. Furthermore, the KOOS analysis showed between group significant statistical differences, but did not reach minimal clinically significant difference. There were no differences in postoperative ROM and VAS. There were no differences in complications between groups. *Conclusions*: To achieve a successful TKA, the precise tool and individualised objective is of great importance. The results suggest satisfactory results after both ra-TKA methods and mTKA. Ra-TKA and mTKA stand for a safe and reliable treatment method for OA. Patients reported excellent alleviation in functional outcomes and the radiological results revealed that the better precision does not necessarily lead to a better outcome. Therefore, ra-TKA does not imply strong enough advantages in comparison to the manual method, especially in terms of cost-efficiency and surgical time.

## 1. Introduction

OA is a non-inflammatory degenerative disorder of joints, which leads to localized decline in hyaline cartilage, osteophytes formation and joint line thinning [1,2]. OA affects knees most frequently and presents with symptoms such as pain, stiffness and loss of function [3]. The treatment modalities are divided into conservative and surgical. TKA is a gold standard as an effective and cost-efficient procedure for end-stage OA, featured by satisfactory implant survivorship, assessed with revision as the primary endpoint above 90% at ten years follow-up [4]. However, up to one-fifth of patients still report dissatisfaction following TKA. Multiple studies have detected the issue of axial or rotational malalignment, and mediolateral and ventrodorsal tilt. Even small displacements of 2.5 mm may further impair the ROM up to 20 degrees [5,6,7]. Multiple attempts in component designing, surgical instrumentation, and surgical techniques have been undertaken to improve radiographic and clinical outcomes of TKA [5,6,7,8].

Therefore, the robotic systems were developed to provide adequate precision, which is required and a crucial element for longevity after TKA. Perfect alignment and implant positioning are considered to be goals for TKA for both clinical and radiological effects and patient- and surgeon-reported excellent performance. Studies focused on measuring failures and complications attribute most of them to a lack of accuracy in implant integration with bones and do not find shortcomings in the technique itself. Thus, ra-TKA allows for a decrease and error threshold of conventional surgery [8,9,10,11]. Ra-TKA allows for more accurate implant positioning when compared to m-TKA [12,13] and less of iatrogenic trauma to the soft tissue envelope [14]. This has been associated with improved early outcomes, such as shorter length of hospital stay, improved ROM and better performance in a shorter period of reconvalescence [15,16,17]. In our institution, semiautonomous robotic systems NAVIO and its next generation CORI are utilized. These are the imageless, handheld robotics, which allow for real-time planning and gap assessment, optimized alignment and balance, robotic-assisted bone resection upon a surgeon control in the most optimized, individualized manner. Constantly, the results do not encourage that ra-TKA systems present better intraoperative and postoperative outcomes and facilitate quicker return to activity than mTKA [18,19,20,21,22,23]. However, the literature lacks systematic reviews or meta-analyses to compare intraoperative (surgical time, tourniquet time, operative time and blood loss) and postoperative (KOOS, VAS, ROM, function, complications, revisions and return to activity) outcomes of the NAVIO versus CORI versus mTKA. The up to date systematic review found currently limited evidence to support the use of ra-TKA. The available studies presented as ‘weak’ evidence to support the novel method and related to UKA only [22]. Hencethere is a lack of well-designed clinical trials addressed to compare ra-TKA and mTKA. Therefore, we conducted a prospective randomized controlled trial with the aim of comparing operative time, blood loss, hospital stay, postoperative complications and clinical outcomes (KOOS, VAS, ROM, and radiographic) between two imageless systems and the manual treatment method for KOA.

## 2. Materials and Methods

This randomized, triple-blinded (Care Provider, Investigator, Outcomes Assessor) clinical trial included 215 patients who were randomly assigned to one of a three groups to undergo TKA. All of the procedures were performed by a single senior surgeon (KM). The groups were divided according the operation technique. These were: NAVIO or CORI robotic-assisted systems or conventional technique TKA performed on patients fulfilling the inclusion criteria. The recruitment took place between 1 December 2021 and 31 July 2022 at Hospital in Ostrow Mazowiecka, Poland. The randomization process was done by the parallel allocation between the three groups using a computer program. While the learning curve with the NAVIO/CORI was not essential in this study, the surgeon (KM) performed 15 robotic-assisted TKAs using the NAVIO system prior to the start of this study. KM is an experienced surgeon with approximately 1500 mTKA surgeries performed. He underwent traineeship with the professional trainers for NAVIO in May 2020 first and afterwards for CORI in November 2021. Taking into account the aforementioned implications, CORI did not require such a learning curve, because it was launched as an updated version of NAVIO by the same company. This study was approved by the Institutional Committee on Human Rights Related to Research Involving Human of the Medical University of Warsaw under the study protocol: KB/109/2020. All patients had been referred to the hospital for TKA to treat primary KOA and were recruited by a research associate and surgeons (JS, JŚ, KM). All patients were informed of the benefits and risks of robot-assisted TKA and were aware of the potential increased operating time. Eligible patients were those deemed suitable for TKA by a senior surgical author (KM) and were aware and agreed to sign the informed consent. The 215 patients were allocated for either robotic-assisted or conventional method procedures on alternate days of their surgery, with 76 assigned to the NAVIO robotic-assisted TKA cohort, 71 to the CORI robotic-assisted TKA cohort and 68 assigned to the conventional manual TKA cohort. Five TKA procedures were performed regularly in 1day. There were no significant demographic and comorbidities differences between the groups (Table 1 and Table 2).

Participants-inclusion and exclusion criteria

The inclusion criteria for treatment with TKA were: (1). Listed for a primary total knee arthroplasty; (2). suffering from KOA involving one or more compartments; (3). aged 18 or over; and (4). patient willing to provide full informed consent to the trial in polish language. Exclusion Criteria: (1). Primary stage of one-sided KOA; (2). severe symptoms in the contralateral knee so as to require staged bilateral knee replacements within 6 months of the primary procedure; (3). fixed flexion deformity of 15 degrees or greater who will require excessive resection of the distal femur; (4). clinically assessed as varus/valgus deformity of 15 degrees or greater; (5). any co-morbidity which, in the opinion of the investigator, is severe enough to present an unacceptable risk to the patient’s safety; (6). inflammatory arthritis; and (7). unable to understand written and spoken Polish.

Study procedures and data collection

**Figure 1:**
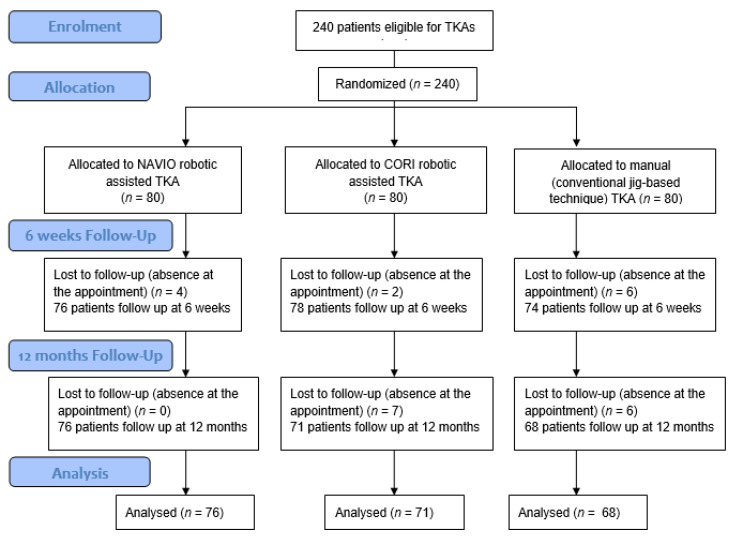
Flowchart of the randomized controlled trial KOOS, ROM in extension, VAS in TKA with the use of ra-TKA and mTKA.

Upon recruiting completion, the medical charts of patients were prospectively completed, consisting of epidemiologic characteristics and details of the surgical procedure and postoperative condition. Figure 1 represents the flowchart of recruiting and follow up. The data collection was carried out at the baseline and after 12 months following the surgery. We have withdrawn from medical files the basic demographic information: (1) age, (2) gender contributions, (3) site of the surgery, (4) ROM, (5) VAS, (6) KOOS, (7) body mass index (BMI), (8) type of anaesthesia, and (9) comorbidities. Prior to the surgery, every patient underwent a blood withdrawal, which is routinely performed for every patient who qualified for the orthopedic surgery. Additionally, we have analyzed perioperative values as: (8) Length of hospital stay, (9) hemoglobin (Hb) levels before and after the surgery in order to assess the blood loss, (10) surgical duration and (11) complications and revision rate. 

All patients underwent TKA procedures with standard perioperative antibiotics prophylaxis with use of 3 doses of 750 mg cefuroxime– preoperatively, 6 and 12 h postoperatively, and standard thrombosis prophylaxis of one dose of 40 mg low-molecular-weight heparin. The day following the surgery, all the patients had to have a control X-ray. During control visits (6 weeks follow up), patients underwent 3D computed tomography scan imaging (CT) to detect the radiographic accuracy of implanted components positioning.

Surgical technique

All surgical procedures were performed by a single experienced surgeon (KM) for ra-TKA and mTKA, respectively. They took place in one hospital with all the patients under epidural anesthesia. A tourniquet was applied and inflated before software registration in the case of ra-TKA and before the skin incision in the case of mTKA. The tourniquet was deflated after wound closure. A standard medial parapatellar quadriceps-sparing incision and approach were used with patellar eversion and patelloplasty. The TKA procedure was standardized and performed using the instrumentation in accordance with the operative technique. 

**Figure 2:**
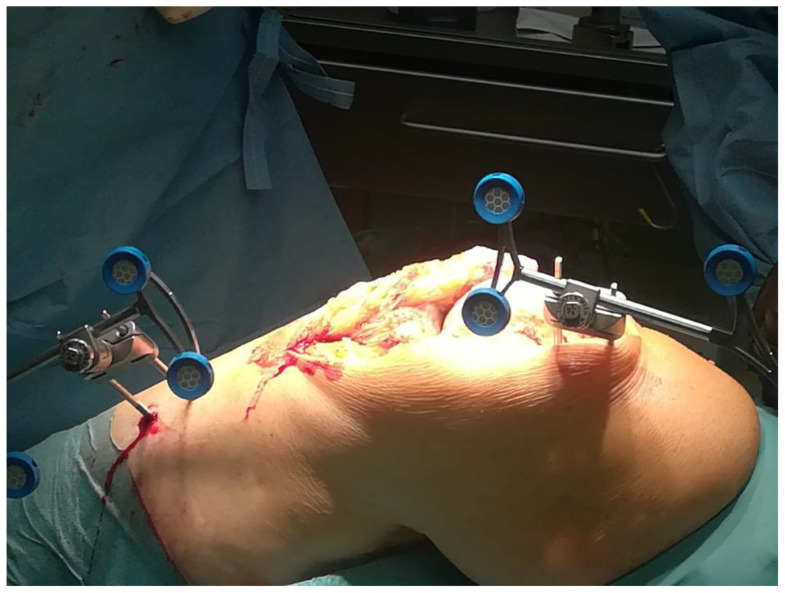
Two-pin bicortical fixation system installed to provide intraoperative TKA assistance using the NAVIO system.

For the ra-TKA groups, additional stabilization pins, trackers and camera setup were installed to allow for navigation markers and bone movement monitors detection of the workspace within the joint. The patient was rigidly connected to NAVIO/CORI software via two transverse stabilization pins in the distal femur and proximal tibia. A detailed registration process was completed by the identification of 4 landmarks on the femur and the tibia (Figure 2). Once registration was completed, the surgeon activated the system and conducted the surgery with robotic assistance described precisely in manuals available at the Smith&Nephew company(London, UK) website [23]. 

Actual surgical technique for the mTKA group consisted of measured resection using an anterior referencing system. Femur cut orientation was prepared using a combination of methods. For a varus knee, the surgeon started with 3 degrees of external rotation based on the posterior condyles. Afterwards, this was checked manually to ensure that this is parallel to the transepicondylar axis. A final confirmation was undertaken by ensuring the Whiteside’s line is perpendicular to the transepicondylar axis. The operation technique was according to the company manual for the implant type. For both ra-TKA cohorts, NAVIO and CORI, the surgical technique consisted of measured resection, respecting gap balancing. Therefore, the method is more of a hybrid technique. The software allows for intraoperative assessment of compartment gaps with fine adjustments using the robotic navigation, all upon surgeon control and incorporating modifications. The robotic system allows for the assessment of the joint intraoperatively through mapping of the articular surface, measurement of the mechanical axis, and measurement of soft tissue tension throughout the full range of motion of the knee. Tibia cut was performed in the coronal plane in the perpendicular axis to the long axis of the tibia. Soft tissue tension, specifically laxity, is measured by applying varus and valgus stress to the knee as it is ranged and allows for the assessment of gap balancing in real time. A surgical plan for component selection and placement, as well as the required bony resection, is created and can be adjusted by the surgeon. Feedback on gap balancing is provided based on the surgical plan and the real-time modifications. In addition to being performed during planning, measurement of soft tissue tension can be repeated during the trialing phase and after soft tissue releases. All patients received a cemented, fixed-bearing prosthesis with metal-bearing polyethylene (Journey II; Smith&Nephew) in the imageless NAVIO and CORI systems and mTKA.

Postoperative care

Patients were discharged after adequate pain control, knee flexion to a minimum of 90 degrees, independent mobilization with the use of crutches, and independent ascent and descent of stairs. All study patients were discharged to home. No patient was discharged to a rehabilitation center or other skilled nursing facility. 

All patients in both groups were prescribed the same standardized postoperative rehabilitation program, with full weight-bearing and active ROM exercises commenced from the day following the surgery. The exercises consisted of bilateral stance (static stance, mediolateral weight shift, multidirectional weight shift), unilateral stance (yoga position with the knee bend on tree position with and without a foam), sit to stand, lunging (static- yoga warrior pose and dynamic- strengthening lunge) and climbing the stairs.

Clinical assessment

Patients were examined during admission for a TKA procedure and followed up postoperatively at 6 weeks and 12 months. Full hospital and clinic medical charts review of demographic preoperative and postoperative knee score measurements were recorded. The primary outcomes of interest were the Knee Injury and Osteoarthritis Outcome Score (KOOS), which assesses patient pain (9 items), other symptoms (7 items), function in daily living (17 items), function in sport and recreation (5 items), and knee related quality of life (4 items). Scores range from 0 to 100 with a score of 0 indicating the worst possible knee symptoms and 100 indicating no knee symptoms. The (KOOS) is an extension of the Western Ontario and McMaster Universities Osteoarthrtis Index (WOMAC), the most commonly used outcome instrument for the assessment of patient-relevant treatment effects in osteoarthritis. The VAS as a pain-measuring scale was utilized in order to obtain the numerical representation of the intensity of the pain bothering the patient throughout the day globally. Additionally, ROM and any postoperative complications were monitored, such as deep vein thrombosis, infection, loosening of implants, fractures, lateral compartment arthritis, and dislocations of the polyethylene component.

Radiographic assessment

**Figure 3:**
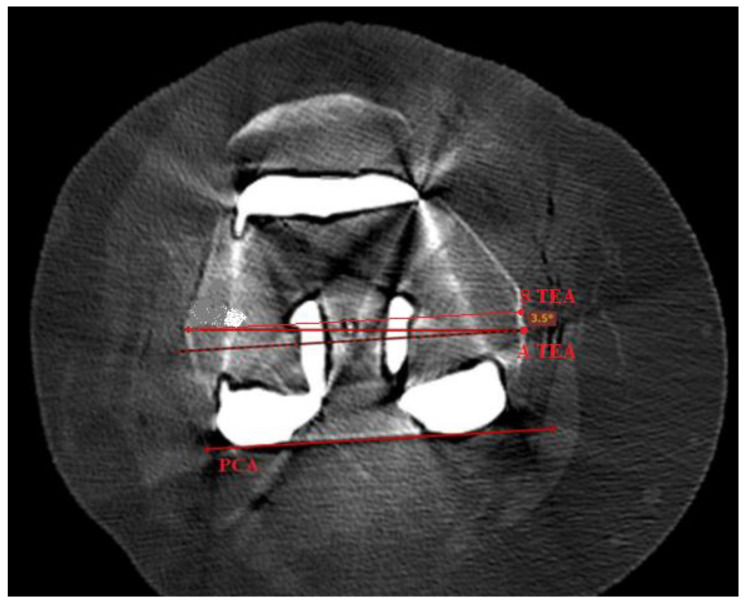
Postoperative TKA CT scan of one of the patients analyzed in the study. It reveals the graphical representation of S TEA and PCA lines, utilized and described in the study methods.

All patients underwent a CT scan at 6 weeks follow up appointments with use of a multislice scanner (General Electric Light Speed Plus; GE Medical System, Milwaukee, WI, USA) to determine the rotational alignment of the components. The CT scan sequence was between 10 cm proximal to the superior pole of the patella and 10 cm distal to the tibial tuberosity and was made in contiguous 2.5-mm slices. Specifically, we measured the anatomic rotational axis on postoperative CT scans in knees of patients undergoing conventional primary TKA (mTKA). We then measured the femoral component rotational alignment optimized by a robotic system (raTKA), which took into account constitutional anatomy of the patient and best functional performance to the posterior condylar axis (PCA) and measured the difference between the femoral component rotation and the actual transepicondylar axis (TEA) (Figure 3). 

Femoral rotation in the following study was defined based on the postoperative CT scans as the angle subtended by a line between the most prominent parts on lateral and medial sites (A TEA) and the line between most posterior parts of the condylar components on the medial and lateral sides of the femur, the so called PCA (Figure 3), according to the Berger protocol [24]. After recognition of both lines of the interest, namely the A TEA and PCA, the line parallel to PCA was subtended upward to reach a common point with A TEA. The angle created by both of the lines was recorded as the femoral component rotational alignment. Internal rotation relative to the PCA was given a negative value and external rotation was given a positive value. All radiographic and CT parameters were measured three times (with a 3-day interval between measurements). Two doctors (A.S. and K.M.) performed these measurements on the CT scans of all knees. An experienced arthroplasty surgeon (P.C) measured 20 randomly selected knee CT scans. A fellowship-trained arthroplasty surgeon (J.S.) also measured 20 randomly selected knee CT scans for the calculation of interobserver agreement and correlation. Intraobserver agreement ranged from 0.94 to 0.97.

Statistical analysis

The sample size was calculated to detect a mean difference in KOOS (0–100) between NAVIO and CORI (ra-TKA) and manual method TKA (mTKA). A power analysis was conducted with type-I error set at 0.05 (α b 0.05) and the type-II error at 0.15 (85% power). The estimated sample size was 65 for each group in order to detect the minimal clinically significant mean difference of KOOS of 6.1 units. Loss to follow-up was estimated to be 20%, which yields a required sample size per group. Data were described using means and standard deviations or median (range) as appropriate for continuous data. The values were checked for normality using Shapiro-Wilk test. Because the distributions were not normal, non-parametric tests were used in statistical evaluation (*p* > 0.05). The baseline characteristics of the patients and cointerventions were compared between the intervention groups using the one-way repeated measures of variance (ANOVA) for dependent values (pre- and postoperative). Differences in means between groups and their respective 95% confidence intervals (CI) were recorded. Kruskal-Wallis ANOVA with Dunn’s post hoc multiple comparisons tests correction method was used to determine any significant differences between postoperative scores between the groups. To determine intraobserver agreement for measurements for all radiographic and CT scan parameters, the chance-corrected kappa coefficient was measured [25]. Intraobserver reliability was almost perfect for both ra-TKA and mTKA. The value of kappa was 0.96 for the ra-TKA NAVIO, 0.97 for the ra-TKA CORI and 0.94 for mTKA. For all of the included analyses, a *p*-value was considered to be statistically significant at *p* ≤ 0.05 and the 95% confidence interval was measured. The results are presented as means (SD), quartiles, and proportions (%). The collected data were analysed using the STATISTICA 13.3 TIBCO Software Inc. statistical package. The statistical analysis was performed by the authors (A.S. and O.A.).

## 3. Results

### 3.1. Demographics

Clinical follow-up of 12 months was available in 215 knees (patients), comprising 76 NAVIO, 71 CORI, 68 mTKA (Table 1). There were no significant differences in patients’ age in the groups (*p* = 0.539), sex (*p* = 0.922) and BMI (*p* = 0.996). The site of the surgery did not appear to differ in a statistically significant way (*p* = 0.478). Mean surgery time was shorter in mTKA with the *p* = 0.003. Hospital stay was not prolonged in any of the groups and did not differ significantly (*p* = 0.447). The blood loss was statistically significant less prominent in NAVIO group, with the difference in Hb level from blood sample: 2.74; CORI: 3.11; mTKA: 2.02; *p* = 0.042). To our knowledge no patient presented with the complication in either of the groups. Patients had comparable comorbidities among the groups, as presented in Table 2. Patient compliance with the allocated treatment and follow-up was 95%, 88% and 85%, respectively, to NAVIO, CORI and mTKA groups. 

**Table 1:** Characteristics of patients who underwent NAVIO CORI robotic-assisted TKA at baseline and intraoperatively.

Characteristics	mTKA (*n* = 68)	NAVIO (*n* = 76)	CORI (*n* = 71)	*p* Value
Age at the surgery (SD)	65 (±8.2)	66 (±7.5)	69 (±6.8)	0.539
Sex Female/ Male (%)	35 (54%)/30 (46%)	42 (64%)/22 (36%)	35 (51%)/34 (49%)	0.922
BMI, kg/m^2^, mean (SD)	26.0 (±3.17)	25.8 (±3.3)	25.5 (±2.9)	0.996
Site of surgery Left/Right (%)	31 (48%)/34 (52%)	34 (52%)/32 (48%)	23 (33%)/46 (67%)	0.478
Epidural anaesthesia (%)	100%	100%	100%	n.a.
Operative time, min, mean (SD)	66.5 (±9)	105 (±8.17)	111 (±11.5)	0.003
Blood loss (Hb level difference before and after surgery), g/dL	2.52 (±1.01)	1.74 (±1.26)	1.51 (±1.12)	0.042
Hospital stay, days, mean (SD)	4.2 (±1.4)	4.4 (±1.0)	4.8 (±1.26)	0.447
Postoperative complications at 1 year, number (%)	0 (0%)	0 (0%)	0 (0%)	-
Revision rate at 1 year, number (%)	0 (0%)	0 (0%)	0 (0%)	-

SD—Standard Deviation. Hb—Hemoglobin.

**Table 2:** Comorbidities among the study cohort.

	mTKA (*n* = 68)	NAVIO (*n* = 76)	CORI (*n* = 71)	*p* Value
Comorbidity, *n* (%)	57 (83.8%)	49 (64.5%)	51 (71.8%)	0.9733
Hypertension	16 (23.5%)	18 (23.7%)	19 (26.8%)	0.0782
Arrhythmias	5 (7.4%)	2 (2.7%)	0	0.8793
Hypothyroidism	5 (7.4%)	3 (3.9%)	6 (8.5%)	0.0812
Diabetes mellitus	13 (19.1 %)	16 (21.1%)	11 (15.5%)	0.0765
Asthma	3 (4.4%)	1 (1.3%)	0	0.5210
Depression	3 (4.4%)	0	1 (1.4%)	0.9854
Systemic lupus erythematosus	1 (1.5%)	0	2 (2.8%)	0.9982
Rheumatoid arthritis	3 (4.4%)	2 (2.7%)	5 (7%)	0.3965
Gout	7 (10.3%)	4 (5.3%)	5 (7%)	0.2135
Psoriatic arthritis	1 (1.5%)	3 (3.9%)	2 (2.8%)	0.0754

### 3.2. Functional Outcomes

The results of the functional and outcome questionnaires are shown in Table 3. All in all, the three groups did not differ statistically significantly according to baseline functional characteristics. Functional outcomes measured by KOOS presented with *p* = 0.16174. The remainder of the preoperative outcome measures were similar between the groups. For the postoperative results, at 12 months there was a statistically significant difference in the improvement in the KOOS, with an 87.05 ± 7.74 increase in score for the NAVIO group compared with a 85.59 ± 8.03 increase for the CORI group and compared with a 81.76 ± 8.95 for the conventional group. After controlling the postoperative outcomes with minimal clinically significant mean difference of 6.1 units for the presenting cohorts for KOOS, the results did not show the minimal difference from a clinical point of view. 

ROM in extension presented with *p* = 0.0902 and ROM in flexion appeared with *p* = 0.5498. Only VAS was measured with a statistically significant difference of *p* = 0.0062. Talking about postoperative results of KOOS, they showed improvement with *p* = 0.0001. ROM showed insignificant results in extension and in flexion of *p* = 0.9867 and *p* = 0.0621, respectively. VAS was measured to be statistically insignificant in the postoperative follow up (*p* = 0.1098). Postoperative outcome measures of femoral component rotation alignment showed the only applicable statistically significant difference (*p* = 0.0013) with the better precision, with a 1.48 ±1.117 degree for the NAVIO group compared with a 1.33 ± 1.012 degree for the CORI group and compared with a 3.15 ± 1.2163 degree for the conventional group. 

Table 4 shows that both ra-TKA groups and mTKA group demonstrated significant improvements in the majority of the outcome scores at 12 months (postoperative values) (*p* < 0.0003–0.3) in comparison to preoperative scores. The outcomes of patients who received treatment with the NAVIO expressed statistically significant improved sores in KOOS (*p* = 0.0109), ROM in extension (*p* = 0.0042), VAS (*p* = 0) and femoral component rotational alignment (*p* = 0.0006). The CORI group progressed with KOOS (*p* = 0.0048), ROM in extension (*p* = 0.0013), VAS and femoral component rotational alignment with *p* = 0.0003 and *p* = 0.0127, respectively. Patients treated with mTKA showed statistically significant improvement in the following scores KOOS, ROM in extension, VAS and femoral component rotational alignment with *p* = 0, *p* = 0, *p* = 0.0491 and *p* = 0.0048, respectively. The scores of ROM in flexion did not yield statistically significant improvement. The comparison of the preoperative and postoperative values of KOOS with minimal clinically significant mean difference of 6.1 units for the presenting cohorts, the results did show the minimal difference from a clinical point of view. In each study cohort, the results exceeded the minimal clinical difference significantly with the pre- to post-operative outcomes presenting, in the NAVIO group: 30.79 to 87.05, in the CORI group: 28.18 to 85.59, in the conventional group: 30.3 to 81.76.

Comparing the groups, Table 5 presents statistically significant KOOS differences between mTKA and NAVIO (*p* = 0.0498) and mTKA and CORI (*p* = 0.0382). Femoral rotational alignment also appeared to differ statistically significantly between mTKA and NAVIO (*p* = 0.0376) and mTKA and CORI (*p* = 0.0011). Otherwise, there were no significant differences in ROM and VAS scores at 12 months between the three groups (*p* = n.s.).

### 3.3. Radiographic Evaluation of Femoral Component Rotational Alignment Postoperatively

Radiographic findings revealed no differences between the two ra-TKA groups but showed improvement in comparison with mTKA. Mean radiographic postoperative femoral rotational component alignment in NAVIO vs. CORI vs. mTKA were 1.48 vs. 1.33 vs. 3.15. These values showed statistically significant difference comparing ra-TKA (NAVIO: *p* = 0.0376; CORI: *p* = 0.0011) with mTKA, but not between the two ra-TKA systems. Table 5 indicates the results of the post hoc Dunn’s multiple comparisons test of performance of each from the therapeutic method in terms of functional and radiographic results. 

**Table 5:** Ra-TKA vs. mTKA postoperative outcomes represented as results of Kruskal-Wallis test and the post hoc Dunn’s multiple comparisons test, showing significance of differences in analyzed values between ra-TKA (NAVIO/CORI groups) and the mTKA group.

Characters	Intervention Group	Intervention Group	Kruskal-Wallis Test		Dunn’s Multiple Comparisons Test
			H	*p*	
KOOS	mTKA	NAVIO	82.232	0.0498	***
	mTKA	CORI	24.672	0.0382	**
	NAVIO	CORI	2.976	0.6459	n.s.
VAS	mTKA	NAVIO	34.567	0.1852	n.s.
	mTKA	CORI	47.160	0.6498	n.s.
	NAVIO	CORI	64.567	0.0783	n.s.
ROM in extension	mTKA	NAVIO	48.322	0.0971	n.s.
	mTKA	CORI	22.548	0.9447	n.s.
	NAVIO	CORI	36.901	0.7115	n.s.
ROM in flexion	mTKA	NAVIO	35.230	0.3491	n.s.
	mTKA	CORI	22.375	0.6948	n.s.
	NAVIO	CORI	3.578	0.0998	n.s.
Femoral Component Rotational Alignment	mTKA	NAVIO	22.497	0.0376	**
	mTKA	CORI	48.345	0.0011	***
	NAVIO	CORI	36.522	0.0935	n.s.

H: H test value; n.s.: non-significant; *p*: significance level; **: *p* ≤ 0.05; ***: *p* ≤ 0.01.

### 3.4. Complications 

**Figure 4:**
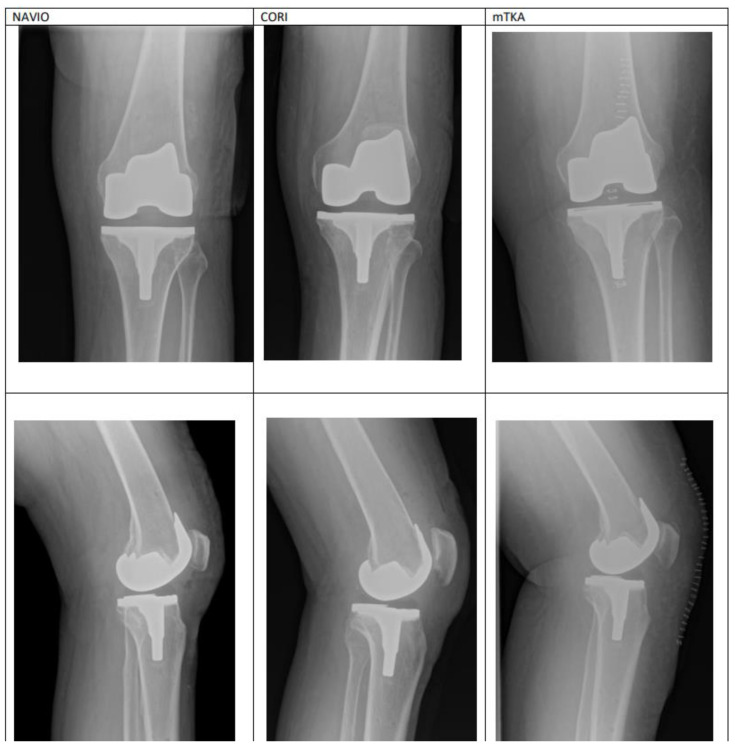
Control entitled X-rays from the day following each procedure: NAVIO and CORI ra-TKA, and mTKA.

There were no between-group differences in terms of the frequency with which complications occurred. Neither group had delayed complications, such as: Infection, patellar dislocation, patellar fracture, supracondylar fracture, peroneal nerve palsy, periprosthetic fracture, thromboembolism, compromised wound healing occurred and no robotic-specific complications such as: Pin site fracture or pin tract infection occured, and no revisions were reported in either group. No deep infection occurred in these knees. No complications were detected on contol X-rays. Figure 4 presents the example of X-ray imaging performed on patients from each group during hospital stay (Table 1).

## 4. Discussion

The introduction of novel methods into TKA is stimulated by increasing the abundance of younger patients affected by KOA particularly. It is suspected to be a collection of distinct subtypes, each with a different etiology and clinical characteristics. KOA can be classified into multiple disease entities, such as: post-traumatic osteoarthritis, age and rheumatological-related OA or depending on genetical factors, family history, sex or finally obesity. Understanding its heterogeneity, as in the case of post-traumatic KOA, can contribute in the development of potential interventions targeted toward individual disease processes [26].

To address patients’ expectations and improve quality of life, advancements are continuously implicated. 

The robotic systems used overall in clinical practice relied on an image-based method. Ren et al. conducted systematic review and meta-analysis studying the clinical performance and patients’ satisfaction after image-based ra-TKA. Patients presented with more precise mechanical alignment and implant position, but no significant differences in the ROM and complication rates, but were at increased risk for adverse events caused by excessive radiation exposure in comparison to mTKA [27]. Systematic review and meta-analysis conducted by Zhang et al. evidenced the superiority of the robotic system in terms of clinical outcomes [28]. However, neither of the referenced papers related only on randomized controlled trials. Yet, Kim et al. reported that there was no significant difference in ra-TKA and mTKA in terms of clinical and radiographic outcomes at 10-years follow-up [29]. These finding confirm that so far the literature yields heterogenous results [30].

To date, there is a paucity of literature comparing the clinical outcomes of ra-TKA and mTKA with the use of NAVIO or CORI, and if it is, it concerns unilateral knee arthroplasty in the majority. However, some studies report on similar performance of both methods in terms of KSS, WOMAC (24-months postoperatively), perioperative measures (hospital stay, infections, and revision rate) and length of the surgery [31,32]. 

**Figure 5:**
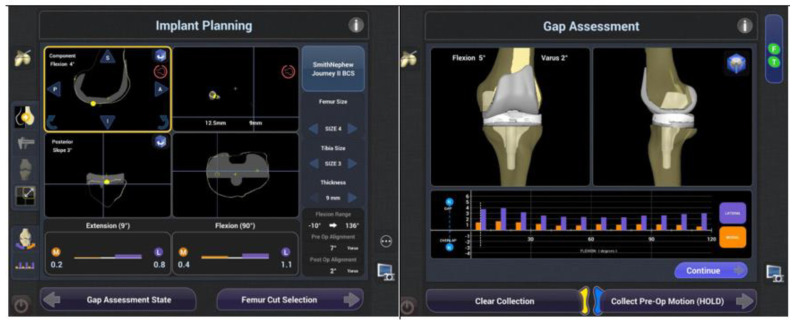
Intraoperative implant planning, utilizing the NAVIO surgical system.

Newer generations of ra-TKA, like the ones presented in this study, demonstrate advancements in ligaments and soft-tissues check-ups in real-time throughout the entire ROM without the preoperative imaging. Figure 5 shows that it simplifies preoperative logistics and utilizes intraoperative planning. Di Benetto et al. found that NAVI0 allows for more precise implantation of the unicompartmental prosthesis than mTKA [33]. 

Our study is in agreement with aforementioned papers and demonstrates statistically significant adequacy in radiographically measured femoral component rotational alignment in the NAVIO and CORI groups. The robotic navigation enables a personalized approach with both NAVIO and CORI and balance the knee within the ligamentous tension. We believe that a modern orthopaedic surgery should consider ligamentous tension. Thus, bone references may be the first step to more reliable and reproducible implant positioning to recreate the joint functioning among different arthritic deformities correction. There are several possible methods for detection of rotational alignment, while in this study we utilized one: surgeon uses bone landmarks of the femur intraoperatively as a reference is shown in Figure 3: transepicondylar axis (TEA): Anatomical (lateral to medial epicondyle) or surgical (medial sulcus lateral epicondyle) axis, which has low reproducibility and reliability levels; posterior condylar line (a very reliable reference); Trochlear AP axis, which is known as the Whiteside line. 

Intraoperatively, the right determination of epicondyles is not always possible. If the reference on lateral condyle is unique on the apex of bony prominence, there are two possible references for the medial condyle: The apex of the medial prominence defines the anatomical TEA (A TEA); and the surgical TEA (S TEA) connects the lateral condyle and the medial sulcus on the femur. The chose A TEA, which was made according to Robertson et al. conclusions, that is, the measurements using the A TEA transepicondylar axis are easier to replicate compared to the S TEA axis [34].

Speaking about the importance of accurate femoral component rotational alignment, Newman et al. found that knees have a high rate of rotational asymmetry, therefore to identify and classify the proper value by the case individually allows for the achievement of the best possible match to the patient’s anatomical variation [35]. Twiggs et al. collected a cohort of 726 patients that appeared with multiple anatomic variations in the knee joint. When applying the standardized reference dogma of 3° external rotation to the PCA, 36.9% of patients would have gained a rotational target greater than ±2° from their TEA [36]. RaTKA systems with the ability for intraoperative measurement of femoral component rotational alignment and ligamentous tension reproduce highly suitable alignment based on the individual anatomy of the patient. However, as is presented in the following study, a better precision did not correlate with the clinically significant difference in KOOS, and with statistically significant difference in ROM, in both flexion and extension. 

Further analysis of our study revealed statistically significant longer surgery time in ra-TKA when compared to mTKA. Despite this, ra-TKA patients present with prolonged surgery time, the blood loss is significantly lower than during mTKA. The surgeon in the present study did not perform more than 15 procedures with NAVIO before enrollment of the patients, where Bell et al. evidenced that learning curve was reached at 29 cases with NAVIO [37,38]. The increase in blood loss is not significant in the ra-TKA cohort and these results stands in the line with previously reported outcomes of avoiding of the intramedullary instrumentation [21,35,39]. Another study attributed it to saving the vascular structures by minimizing soft-tissue trauma and performing more precise bone cutting [40]. Paradoxically, Song et al. evidenced a significantly lower mean blood loss (568.6 mL vs. 816.0 mL) despite a larger mean incision length (15.2 cm vs. 13.0 cm) in ra-TKA than mTKA using a medial para-patellar approach. One should note that mean blood loss is multi-factorial, including surgeon, peri-operative and patient specifics [41].

Despite the aforementioned learning curve, the long-lasting surgery time is also associated with using the robot (placement and removal of pins, registering of the joint with the robotic system, and intraoperative planning). The average difference in operative time between the two ra-TKA cohorts ranged from 38.5 to 44.5 min. Increased operating time has been correlated with an increased risk of infection, and an operative duration of 127 min for a TKA procedure has been described as a critical operative duration in terms of infection risk, so it was not reached in any case of present study [42]. There are studies that demonstrate that after a number of surgeries, the surgical time is almost the same with the use of ra-TKA as it is in mTKA. The goal of the novel technologies despite of an enhancement of the results, is to be comparable to manual procedure [43].

Furthermore, functional outcomes (ROM, VAS) present statistically significant improvement after all the procedures. KOOS outcomes scores present improvement in groups of patients, operated with NAVIO and CORI. Onggo et al. conducted the only one systematic review with the mentioning of TKA with NAVIO and their major conclusion underline that both ra-TKA and mTKA are reliable and safe procedure for OA treatment. However, ra-TKA is capable of achieving superior alignment in multiple axes, lower mean blood loss as well as marginally better clinical outcomes than mTKA [40]. The novel imageless, ra-TKA systems are highly accurate with respect to component positioning in coronal plane, mechanical alignment and patellofemoral kinematics as compared to conventional TKA [40,41,42]. These papers yield partially similar results in comparison to our study. KOOS outcomes showed statistical difference in outcomes between groups, however, the minimal clinical relevance was found not significant. 

The efficiency of TKA compared with conservative strategies in a management of KOA has been proven [44]. However, the debate continually arises over the best type of prosthesis and most adequate stage of KOA to perform knee replacement. Many authors are UKA enthusiasts because of preservation of bone stock, faster recovery, lower overall cost, reduced morbidity, better functional outcome because of more normal knee kinematics, and subjective feeling of a more natural knee [45,46,47]. However, the main problem is the higher revision rate, particularly in younger patients in comparison to TKA [47,48].

The type of implant was found to be a predictor of improvement in quality of life postoperatively. Siviero et al. suggest that surgery represents a valid approach to severe OA at any age. Furthermore, a comprehensive assessment can help to identify risk and protective factors for better function outcomes and improved quality of life. Moreover, this study suggests that TKA brings the most satisfactory results in severe KOA pain, particularly in obese patients, irrespective of age and OA stage [49].

Our study faces some limitations. Firstly, the surgeon did not reach the learning curve, while it was ongoing. Secondly, this study was conducted with a short follow up period of 12 months. Thirdly, further study should assess cost-effectiveness of the operation and focus on radiographic evaluation more precisely than only assessing a single value, as it is in case of this study. Fourthly, in our study, the patients were not informed whether they are going to be assigned to a mTKA, NAVIO or CORI group. All the patients from each of the three groups were informed of the possible complications of both kind of surgeries (mTKA, raTKA). Obviously, after the surgery they were aware of wearing additional scars on the skin from pin incisions, however they were further not informed of the particular robot that was involved in the individual surgery. 

Hence, patients from the ra-TKA groups could have been providing better patient-reported outcome measures than those allocated to mTKA. Patients from the NAVIO and CORI group were blinded, but to blind the patients from mTKA would demand the performance of excessive pointless incisions of the skin, simulating pins fixation, which would result in possible increased infection risk, cosmetic defect and is generally ethically inappropriate. 

We cannot exclude from the list of limitations, that in this study we only included the femoral component rotational alignment from radiographic evaluation. The remaining radiographic evaluations are the measures analysed together with remaining clinical outcomes, registered in the study protocol in clinicaltrials.gov in study protocol NCT04611815, namely biomechanical motion and walking outcomes, being a topic of other manuscript. We aimed to include this parameter only in the following study, as it rigorously agrees with the discussed ra-TKA advantage over the mTKA. The femoral component rotational alignment do not influence the mechanical limb axis, as the other do so. Hence, we hypothesise that those measure outcomes correlate with the biomechanical parameters and demand separate discussion. 

Our cohort consisted of patients with multiple comorbidities and severe obesity. They have used the available conservative treatment methods for knee OA, including weight loss, but the efforts failed. Due to multiple cofactors, they had prominent osteoarthritic deformities, therefore the robotic assistance seemed to bring highly individualized and an optimal solution for the replacement and recruiting the joint function. The significant improvement in functional outcomes and pain is visible. Ra-TKA with NAVIO/CORI systems stand for an effective technique that requires non prolonged hospital stay, relatively short length of surgery and quick patients’ recovery with excellent clinical results and patients’ satisfaction. The thorough observation of the study cohort revealed the conclusions of excellent improvement in VAS, KOOS and ROM as well as accurately optimized femoral component rotational alignment in all groups, yielding the results of mTKA and ra-TKA being effective treatment modalities for patients with OA. The results suggest the satisfactory results after both ra-TKA methods and mTKA. Patients reported excellent alleviation in functional outcomes and the radiological results, revealed that the better precision does not necessarily lead to a better outcome. Therefore ra-TKA does not imply strong enough advantages in comparison to manual method, especially in terms cost-efficiency and surgical time. It reveals that to be precise and close to the preplanned objective is of a great importance in order to achieve a satisfactory patient- and surgeon—reported outcomes. Ra-TKA and mTKA stand for a safe and reliable treatment method for OA.

## Figures and Tables

**Table 3:** Results of clinical and radiographic outcomes preoperatively and at 12 months follow up from the three groups.

Variable	Time	Ra-TKA NAVIO *n* = 76	Ra-TKA CORI *n* = 71	Conventional TKA *n* = 68	*p* Value
KOOS	Preoperative results	30.79 ± 11.46	28.18 ± 8.45	30.3 ± 10.00	0.16174
	Postoperative results	87.05 ± 7.74	85.59 ± 8.03	81.76 ± 8.95	0.0001
ROM in extension	Preoperative results	6.5 ± 5.5	5.7 ± 3.2	6.2 ± 5.0	0.0902
	Postoperative results	1.5 ± 3.8	1.8 ± 1.7	1.5 ± 1.3	0.9867
ROM in flexion	Preoperative results	111.2 ± 10.4	113.1 ± 16.7	114.8 ± 11.3	0.5498
	Postoperative results	126.3 ± 14.2	132.1 ± 9.0	124.3 ± 12.6	0.0621
VAS	Preoperative results	7.4074 ± 1.3531	8.1152 ± 1.1314	8.23 ± 1.256	0.0062
	Postoperative results	2 ± 1.3598	2.39 ± 1.012	2.12 ± 1.23	0.1098
Femoral Component Rotational Alignment	Postoperative results	1.48 ± 1.117	1.33 ± 1.012	3.15 ± 1.2163	0.0013

**Table 4:** Results of clinical and radiographic outcomes for dependent values in intervention groups.

Intervention Group	Variable	Preoperative Values	Postoperative Values	*p* Value
Robotic-assisted TKA NAVIO *n* = 76	KOOS	30.79 ± 11.46	87.05 ± 7.74	0.0109
	ROM in extension	6.5 ± 5.5	1.5 ± 3.8	0.0042
	ROM in flexion	111.2 ± 10.4	126.3 ± 14.2	n.a.
	VAS	7.4074 ± 1.3531	2 ± 1.3598	0
	Femoral component rotational alignment	-	1.48 ± 1.117	0.0006
Robotic-assisted TKA CORI *n* = 71	KOOS	28.18 ± 8.45	85.59 ± 8.03	0.0048
	ROM in extension	5.7 ± 3.2	1.8 ± 1.7	0.0013
	ROM in flexion	113.1 ± 16.7	132.1 ± 9.0	0.115
	VAS	8.1152 ± 1.1314	2.39 ± 1.012	0.0003
	Femoral component rotational alignment	-	1.33 ± 1.012	0.0127
Conventional TKA *n* = 68	KOOS	30.3 ± 10.00	81.76 ± 8.95	0
	ROM in extension	6.2 ± 5.0	1.5 ± 1.3	0
	ROM in flexion	114.8 ± 11.3	124.3 ± 12.6	0.3117
	VAS	8.23 ± 1.256	2.12 ± 1.23	0.0491
	Femoral component rotational alignment	-	3.15 ± 1.2163	0.0048

## Data Availability

The data that support the findings of this study are available from the corresponding author—OA, upon the reasonable request.
